# What clinicians should know about the contribution of modern behavioral genetics to psychiatric problems

**DOI:** 10.1017/S0033291725000273

**Published:** 2025-03-13

**Authors:** Robert Plomin, Evangelos Vassos

**Affiliations:** Institute of Psychiatry, Psychology & Neuroscience, King’s College London, London, UK

The question of the relative contributions of nature and nurture to psychiatric problems has been the target of research for more than a century. The last quarter of the 20th century witnessed a shift from environmentalism to a more balanced view that recognizes genetic as well as environmental influences. This research, largely based on twin and adoption studies, revealed radical findings about the environment as well as genetics because it untangled the threads of nature and nurture normally woven together in the fabric of development. Since the turn of the 21st century, the DNA revolution has dramatically accelerated the pace of discovery (Plomin, 2023).

The goal of this editorial was to consider what clinicians should know about the contribution of modern behavioral genetics (including psychiatric and psychological genetics) to psychiatric problems. It is our impression that most clinicians are at least vaguely aware of some of these discoveries, but the blizzard of technical reports can make it difficult to see the implications of these findings from the practical perspective of clinicians. This is an editorial rather than a comprehensive review, but we include some key references. Books that cover most of these topics are available for more detailed reference (Flint et al., 2020; Knopik et al., 2017; Plomin, 2019; Turkheimer, 2024).

## Inherited DNA differences account for about half of the differences between people with psychiatric problems

We doubt that any ‘blank-slaters’ still exist, but the magnitude of genetic influence does not seem to be fully appreciated. Heritability of psychiatric problems, like all behavioral dimensions and disorders, is about fifty percent, meaning that on average in the population about half of the differences between people with psychiatric problems are due to inherited DNA differences (Polderman et al., 2015). Heritability is greater for some disorders than others – for example, heritability is greater for schizophrenia and bipolar disorder and less for major depressive disorder – but the significance for clinicians lies in the huge effect size of genetic influence. Rarely in psychiatry do we find factors that account for five percent of the variance, whereas inherited DNA differences can account for fifty percent. Recognizing that inherited DNA differences are such a major systematic cause of psychopathology is an antidote to any lingering environmentalism that assumes we are what we learn.

Given the normal range of experiences, some individuals are inherently more vulnerable to mental illness, more resistant to therapy, and more likely to return to their genetic trajectory after therapy (Mundy et al., 2024). These implications may seem to be too general to be of practical use for clinicians, but general perspectives often have the widest applications. For example, instead of assuming that correlations between a person’s psychopathology and experiences with their parents are caused by their upbringing, it may be useful to consider the fact that parents and their offspring, in addition to their shared environment, share fifty percent of inherited DNA differences. The most far-reaching implication of finding that psychopathology is substantially heritable is that it has become a target for the DNA revolution, which will be transformative for clinicians, as discussed later.

It is important to emphasize what this finding of fifty percent heritability does *not* mean for clinicians. Heritability refers to the average influence of genetic and environmental sources of *differences* between individuals, not to a single individual. Most importantly, genetic influence does not imply hard-wired determinism that challenges the utility of therapy. Genetic research describes *what is* in a population, and the average influence of genetic and environmental factors that exist in that population; it does not limit *what could be.* As an extreme example, if psychopathology were 100 percent heritable, a therapeutic intervention could nonetheless have a major impact on symptom reduction, course of illness, and quality of life. A well-known example is the recessive mutation that causes phenylketonuria, whose effects can be ameliorated by dietary intervention that begins early in life. In addition, like all descriptive statistics, heritability can only describe the samples investigated, and the samples used in genetic research do not usually include the environmental extremes of severe neglect and abuse or the genetic extremes of single-gene mutations.

## Environmental influences are not nurture

The rationale for twin and adoption studies is to disentangle nature and nurture which are intertwined in families whose members are related genetically as well as environmentally. After six decades of research on psychopathology, it became clear that genetics almost completely accounts for family resemblance. This body of research provides the best evidence we have for the importance of environmental influences because heritability is only fifty percent, but it reveals that what runs in families is nature not nurture. That is, whatever environmental influences on psychopathology are, they hardly make family members any more similar than individuals in different families (Knopik et al., 2017). In other words, growing up in the same family does not make siblings similar, which is why it is called *nonshared environment* (Plomin & Daniels, 1987).

Why are children growing up in the same family so different? The nonshared environmental origins of psychopathology could include, for instance, accidents and illnesses and relationships such as interactions with teachers, friendships, and intimate relationships. Three decades of research trying to identify these nonshared environmental factors – such as using the powerful design of asking why identical twins differ – have largely come up empty-handed (Kendler & Halberstadt, 2013). Looming large is the radical possibility that nonshared environmental influences are random in the sense of being unpredictable, a topic receiving increasing attention throughout the life sciences as noise generated by the complexity of biological systems (Plomin, 2024).

The importance of nonshared environment requires a difficult conceptual shift for some clinicians from blaming psychopathology on nurture, to thinking about environmental factors that make children growing up in the same family different. We reiterate that this research addresses the normal range of environmental and genetic variation, not the extremes of severe neglect and abuse or rare genetic mutations. If nonshared environmental factors are random, the implications are enormous, beginning with letting go of reductionistic, deterministic beliefs and embracing randomness, realizing that life experiences are more a matter of chance than choice (Davey Smith, 2011). But embracing randomness does not mean accepting helplessness – as noted earlier, genetic research describes what is rather than what could be. In addition, accepting that life experiences are more a matter of chance than choice could help combat the stigma associated with mental illness. A specific goal could be to build up resilience to buffer people, especially genetically vulnerable people, against the inevitability of randomness.

## Most ‘environmental’ measures show substantial genetic influence

Genetic research has revealed another important finding about the environment: ‘environmental’ measures widely used in psychopathology research show substantial genetic influence (Plomin & Bergeman, 1991). Across a wide range of measures such as parenting, life events, and social support, the average heritability is about 25 percent (Kendler & Baker, 2007). The reason why measures labeled as environmental show genetic influence is that they do not assess the environment ‘out there’ independent of us. They assess how we select, modify, and create our experiences, as well as how we report our experiences. This is how genetic propensities permeate our experiences (Plomin, 1994).

Knowing that genetics pervades both environmental measures and psychopathology, it should not be surprising that correlations between them are substantially mediated genetically. Although all clinicians know that correlation does not imply causation, it is difficult to resist the temptation to interpret such correlations as causal, especially correlations between parenting and children’s psychopathology. However, the first application of multivariate genetic analysis to the correlation between maternal negativity and adolescent children’s antisocial behavior found that two-thirds of the correlation could be attributed to genetic factors (Pike et al., 1996). More than a hundred subsequent twin and adoption studies have shown similar results (Plomin et al., 2016), and these results are being confirmed using DNA to index genetic risk. For example, there is evidence that high genetic risk for a variety of mental disorders may affect an individual’s choice of residence (Maxwell et al., 2021).

In other words, correlations between experiences and psychiatric problems can partially be genetic effects in disguise. Clinicians can help people realize how they create correlations between their genetic propensities and their experiences so that they can anticipate and disrupt these correlations. For example, for patients with a genetic predisposition to dependence, it may be important to avoid life choices that increase their exposure to alcohol or drugs. It is important to highlight that genetic influence is not synonymous with determinism or helplessness, and there is substantial scope for impactful therapeutic interventions by clinicians.

## Some of the many DNA differences responsible for heritability have been identified

The DNA revolution has made the abstract concept of heritability concrete by identifying inherited DNA differences that contribute to the heritability of psychopathology (Abdellaoui et al., 2023). This research has proven that the heritability of psychopathology is not due to a single gene or even a handful of genes but rather to thousands of DNA differences most of which have incredibly small effect sizes (Visscher et al., 2021). This finding shifts thinking about genetic influence from hard-wired programming to probabilistic propensities.

The biggest effect sizes of common genetic variants are much smaller than anyone anticipated: Risk ratios are barely greater than 1.0 for case-control studies, accounting for less than .01% of the variance in liability. We should note that rare genetic variants, such as copy number variants (CNVs), can have a dramatic effect on individuals, but they only affect a tiny fraction of the population and their contribution to the overall variance explained is small (Owen et al., 2023). Nonetheless, some rare genetic variants are actionable, and a recent report by the Royal College of Psychiatrists recommends testing for CNVs in patients with schizophrenia who have co-occurring conditions or young people with certain neurodevelopmental disorders (Royal College of Psychiatrists College Report CR237, 2023).

## Polygenic scores can significantly predict psychiatric problems

These tiny effects of DNA differences on psychiatric problems can be aggregated into polygenic scores that can predict psychopathology. Polygenic scores derived from case-control genome-wide association analyses of millions of inherited DNA differences can currently predict about 8% of the variance of liability to schizophrenia (Trubetskoy et al., 2022), 8% for bipolar disorder (O’Connell et al., 2023), 6% for major depression (Adams et al., 2025), 5% for attention-deficit/hyperactivity disorder (Demontis et al., 2023), and 2% for autism spectrum disorder (Grove et al., 2019).

Although the predictive power of current polygenic scores is modest and of minimal use clinically in the general population, it is expected that more predictive polygenic scores will be informative at different points in the disease trajectory (Lewis & Vassos, 2022; Murray et al., 2021). It should be noted that environmental predictors of psychopathology, such as parenting, explain less variance, especially after controlling for genetic mediation. Moreover, current polygenic scores can make useful predictions at the extremes. For example, individuals in the top one percent of polygenic scores for schizophrenia are nearly 40 times more likely to be diagnosed as schizophrenic as compared to individuals in the lowest centile (Trubetskoy et al., 2022). The predictive power of polygenic scores will increase as sample sizes increase and scoop up more tiny effects and as whole genome sequencing of all 6 billion base pairs of DNA captures rare variants not assessed using the current genotyping technology of DNA microarrays that only genotype common variants.

As polygenic scores become more predictive, they will transform clinical work. Their unique benefit is that they are causal in the sense that nothing in the brain, behavior, or environment changes the DNA sequence inherited in the first cell with which our lives began, and the same DNA is found in the trillions of cells of our bodies. This means that polygenic scores can be used as an early warning system able to predict just as well from infancy as from adulthood. Prediction permits prevention, and an ounce of prevention may be worth a pound of cure. In addition, polygenic scores are being used in research to predict responses to interventions and treatments. If successful, this research could produce polygenic scores to help clinicians personalize their practice (Lewis & Vassos, 2022; Polygenic Risk Score Task Force of the International Common Disease Alliance et al., 2021).

Clinicians will increasingly be confronted with the results of DNA testing as millions of people have already paid about £100 to direct-to-consumer companies to obtain their DNA results. Genotyping needs to be done only once, which can be used to construct any of the dozens of polygenic scores now available for psychopathology. UK Biobank has genotyped 500,000 people who have provided information about psychiatric problems and permitted access to their NHS records. Another ongoing program in the UK called Our Future Health is genotyping five million people with access to their NHS records. Eventually, all infants will be genotyped at birth, not just for the handful of single-gene mutations for which they are currently genotyped, but for the whole genome to predict and prevent problems rather than waiting until problems appear and then trying to cure them.

Clinicians should know that extant polygenic scores are less predictive in ancestries other than the northern European and American samples from which the polygenic scores were derived. However, research in progress on more diverse samples, such as Our Future Health in the United Kingdom and All of Us in the United States, will improve the transferability of polygenic scores (Lewis & Vassos, 2022).

## Current diagnostic classifications bear little resemblance to the genetic foundations of psychiatric problems

Genetic research has revealed two findings that undercut the diagnostic foundations of psychiatry. The first is that the genetic architecture of psychiatric problems differs profoundly from diagnostic classification systems based on symptoms. The most dramatic illustration involves schizophrenia and bipolar disorder. Based on symptoms, these disorders were assumed to be etiologically distinct, but when the first genome-wide associations studies were conducted, many of the same DNA differences were associated with both disorders. Subsequent research has shown that the genetic correlation between schizophrenia and bipolar depression is 0.68, meaning that two-thirds of genetic effects overlap between the two diagnoses. Another surprising finding is that PTSD is highly correlated genetically with MDD (0.75), anxiety (0.58), and ADHD (0.78). Substantial genetic correlations between other diagnoses are less surprising, for example, between MDD and anxiety (0.87), anorexia and OCD (0.46), and ADHD and autism (0.38) (Grotzinger et al., 2022). Because the genetic correlations are less than 1.0, there are of course genetic differences between disorders as currently diagnosed. For example, the total burden of rare variants and specific CNVs are known to differ between schizophrenia and bipolar disorder (Owen et al., 2023; Royal College of Psychiatrists College Report CR237, 2023).

Even beyond these genetic clusters, there is a positive manifold among 11 disorders that have been the target of large genome-wide association studies, which provide the data for these analyses. Of the 55 pairwise genetic correlations between the 11 disorders, the average genetic correlation is 0.28. This genetic overlap among disorders is reflected in substantial phenotypic comorbidity among disorders, which yields a general transdiagnostic factor of general psychopathology, called p because it is analogous to g, the general factor of cognitive ability. Genetic research shows that this ubiquitous comorbidity indexed by p is mostly genetic in origin (Selzam et al., 2018).

These genetic correlations among disorders that currently occupy different positions in psychiatric taxonomies suggest a genetic architecture of psychiatric problems that differs greatly from current diagnostic classifications. Genetic evidence for p implies greater vulnerability to psychopathology in general, not just to specific disorders. Clinicians will be aware of the porousness of diagnoses but perhaps not to the extent indicated by genetic p, which may prompt thinking about transdiagnostic interventions and treatments (Plomin, 2022).

The second finding challenges the very notion of diagnosing disorders, if what is meant by disorders is etiologically distinct categories, as engrained in the medical model of illness. To the contrary, genetic research indicates that there are no qualitative disorders, just quantitative dimensions (Plomin et al., 2009). Individuals diagnosed with cases of a disorder do not have mutations unique to their diagnosis. As noted earlier, there are thousands of DNA differences responsible for heritability; people diagnosed with a disorder are likely to have more of the DNA differences associated with the disorder than controls, but the difference is quantitative. Polygenic scores are perfectly normally distributed in the population, with no hint of bimodality separating cases from controls. More direct evidence indicating that disorders are dimensions comes from genetic correlations greater than 0.90 between diagnosed disorders and dimensional measures of psychiatric problems (Taylor et al., 2019).

The clinical utility of discovering the importance of genetic p and the dimensional nature of disorders might seem limited by the practical dictates of the diagnostic regime, but we predict that this regime will eventually be overthrown. This is not to deny that there are psychiatric problems, but it is too much of a simplification to reify diagnostic disorders as etiologically distinct and dichotomous rather than overlapping dimensions.

## What clinicians do not need to know

Two issues that affect the interpretation of polygenic scores are the focus of much current genomic research. One topic is called genetic nurture because it involves indirect environmental transmission of genetic effects from parent to offspring (Kong et al., 2018). The other issue is that polygenic score prediction in the population can be due to differences between families, such as population stratification (e.g., ancestry, socioeconomic status), which can be considered an indirect effect in the sense that polygenic score prediction in the population is not reflected within families (Howe et al., 2022). These are complicated issues, but fortunately they can be safely ignored by clinicians because genetic nurture and between-family effects are found mostly for cognitive traits, not for psychopathology (Lin et al., 2024).

## DNA

We were motivated to write this editorial because the radical findings from genetic research warrant more attention from clinicians. These findings begin with the discovery that inherited DNA differences are the major systematic source of mental health problems. Environmental causes of psychopathology are not due to nurture, systematic effects of the family environment, but rather to nonshared experiences that appear to be unpredictable. What looks like systematic effects of the environment are genetic effects in disguise.

These findings came from the first half of the modern era of behavioral genetics, which is said to begin in 1960 with the publication of an eponymous textbook (Fuller & Thompson, 1960). The DNA revolution began in the 1990s when it became possible to genotype inherited DNA differences directly and assess their associations with common disorders and complex traits. Polygenic scores can already predict psychiatric problems significantly albeit modestly. As polygenic scores become more powerful predictors of genetic risk, they will transform clinical work, serving as an early warning system for prevention and as predictors of treatment response. A 2023 report calls for genomic testing being embedded into clinical care pathways and identifies the All Wales Psychiatric Genomics Service as an exemplar service design and delivery of genetic testing (Royal College of Psychiatrists College Report CR237, 2023). An unexpected way in which the DNA revolution is already transforming clinical work is to show that current diagnostic classifications bear little resemblance to the genetic architecture of psychiatric problems.

The acronym *DNA* has been used by clinicians to mean that the client *did not attend.* We hope that clinicians will join the DNA revolution so that DNA comes to mean *deoxyribonucleic acid.*
